# Case Studies of Fish Habitat Compensation in Eeyou Istchee: Compensation Projects Prioritize Facility over Effectiveness: Fish Habitat Compensation in Eeyou Istchee: Is Trying Enough?

**DOI:** 10.1007/s00267-025-02276-1

**Published:** 2025-09-15

**Authors:** Kathleen D. W. Church, Adriana Raquel Aguilar-Melo, Hugo Asselin, Katrine Turgeon

**Affiliations:** 1 Université du Québec en Outaouais, Institut des Sciences de la Forêt tempérée, Ripon, QC Canada; 2 Université du Québec en Abitibi-Témiscamingue, School of Indigenous Studies, Rouyn-Noranda, QC Canada

**Keywords:** Fish habitat compensation, Industrial development, Eeyou Istchee, No Net Loss (NNL), Indigenous Knowledge (IK)

## Abstract

Industrial activity, particularly hydropower and mining projects and their associated road networks, are prevalent in Eeyou Istchee, the traditional home of the Crees in the James Bay region of Northern Quebec. Since the mid-1980s, industry proponents must outline plans for fish habitat compensation in order to receive authorization from Canada’s Department of Fisheries and Oceans to engage in any development activity that will result in the harmful alteration, disruption or destruction of fish or fish habitats. The goal of these fish habitat compensation projects is No Net Loss of Canada’s fish habitat productivity, with fish habitat compensation serving as a compromise between continued industrial development and the preservation of Canada’s fisheries resources. In this paper, we outline five recent industrial development projects and their associated fish habitat compensation projects in Eeyou Istchee. These projects include a hydropower project, two mining projects, a road extension project, and the repair of two existing roads. The inclusion of Cree traditional knowledge, the impacts of the development projects on fish and fish habitats, the avoidance and minimization measures taken during the habitat compensation work, and the implemented fish habitat compensation projects are summarized and compared for each project. The priority for these five fish habitat compensation projects was their structural integrity and potential ability to function as designed, rather than any proven beneficial effects on fish reproduction and fish population dynamics. In cases where fish populations continued to decline despite the habitat compensation projects, nothing further was done. Proponents were only held accountable for the completion of the planned compensation work, but not for the consequences of their fish habitat compensation projects.

## Introduction

Freshwater habitats provide ecosystem services to billions of people, including marginalized and vulnerable communities (Lynch et al. 2016). However, freshwater ecosystems are currently experiencing rapid population declines. Alarmingly, populations of monitored freshwater species worldwide, including fish, have decreased by 84% since 1970, indicating a much steeper drop compared to terrestrial and marine ecosystems (WWF 2020). Many declines in fish populations are a direct response to the harmful alteration, disruption and destruction (i.e., HADDs) of freshwater habitats due to various industrial development activities, for example hydropower, mining, forestry and their associated infrastructure, as well as road development (Strayer and Dudgeon 2010; Winemiller et al. 2016; Reid et al. 2019; WWF 2020).

In Canada, the solution put forward to conserve fish and their habitats while allowing the continuation of natural resource extraction is through fish habitat compensation. Since 1985, the rules for the protection of fish and fish habitat in Canada and the legislative management of Canadian fisheries, have been established via the federal Fisheries Act. The Policy for the Management of Fish Habitats was implemented in 1986 to ensure that for most major development projects, authorized HADDs were to be offset by legally binding fish habitat compensation, based on the goal of No Net Loss (NNL; Quigley and Harper 2006a; DFO Fisheries and Oceans Canada 2025). For any HADD to be allowed, industrial development companies first need to receive authorization (i.e., a permit) from the federal government’s Department of Fisheries and Oceans (DFO) via Section 35(2) of the Fisheries Act, including their plans for fish habitat compensation (Quigley and Harper 2006a; Favaro et al. 2012; Favaro and Olszynski 2017). However, it has proven difficult for NNL of fish and fish habitats to be achieved in reality, either in terms of fish productivity (DFO Fisheries and Oceans Canada 2019) or fish habitat (DFO Fisheries and Oceans Canada 2025). Fish productivity is certainly not the only indicator of aquatic ecosystem health (e.g., Lapointe et al. 2014), and although fish population size and reproduction are common measures used to determine the success of a given habitat compensation project, they do not provide a full picture of productivity (MacLeod et al. 2022; Ohlberger et al. 2022). Most studies of habitat compensation determine NNL based on the total habitat area lost through development and then gained through habitat compensation, rather than on science-based assessments of the productive capacity of these habitats before and after development, as this is more difficult and time-consuming to determine (Quigley and Harper 2006b). As a result, HADDs tend to be larger than authorized for at least half of development projects (Favaro and Olszynski 2017; Harper and Quigley 2005a), and a substantial proportion of these compensation projects fail to achieve NNL (Harper and Quigley 2005a).

Nevertheless, despite the continued NNL policy implementation failure to effectively protect fish and fish habitats from industrial development, the Fisheries Act was changed in 2012 to narrow the scope of fish habitat protection despite strong opposition to these changes from the scientific community, Indigenous groups, and from general society (Olszynski 2015). However, these lost protections were restored by the federal government in 2019, alongside additional provisions to the Fisheries Act intended to facilitate reconciliation efforts with Indigenous peoples (Government of Canada 2019). In this version of the Fisheries Act, Indigenous knowledge (IK) is now more present in the policy, as it is considered more in decision-making regarding fish habitats, while increased Indigenous representation on advisory panels for industrial development is also promoted (Government of Canada 2019). However, the exact phrasing used in the Act (i.e., “shall consider”, “may consider”) indicates that the extent to which IK is used or considered for any given project is still subject to the Minister’s decision.

Cree knowledge encompasses management practices, knowledge, and beliefs, as is typical of Indigenous and traditional societies worldwide (Asselin 2015; Berkes 2017); this knowledge also reflects the complex interactions between humans and nature (Nelson and Shilling 2018). More recently, IK has become increasingly recognized for its complementarity with scientific knowledge (SK) in improving conservation and restoration practices (Uprety et al. 2012; Albuquerque et al. 2021; Bélisle et al. 2021). Scientific evidence shows that the combination of IK and SK can better guide and prioritize biodiversity conservation actions (Moller et al. 2004; Campos-Silva et al. 2018; Shokirov and Backhaus 2020). Cree knowledge could thus be particularly helpful to inform compensation projects for fish habitats in Eeyou Istchee (see Church et al. n.d.), as well as to better integrate Indigenous concerns regarding the effects of industrial activities and provide meaningful compensation for habitat loss (Reid et al. 2021).

Eeyou Istchee (Cree: ), located in Northern Quebec, Canada, is the traditional territory of the Cree Nation. In Eeyou Istchee, healthy waterways and aquatic biodiversity are integral parts of overall ecosystem health and of Cree cultural wellbeing, while freshwater fish are a dietary staple for the Crees. This territory includes some of the lakes and rivers that drain into James Bay and Hudson Bay, and is home to more than 18,000 Crees belonging to ten different communities (Cree Nation Government 2015). Eeyou Istchee covers ~400,000 km^2^ and includes over 300 family hunting grounds, known as traplines, that are occupied, used and managed by Cree families for cultural practices (Awashish 2018). This territory embraces a wide range of environments and freshwater fish habitats. For the Cree people, all of this land is sacred, including the waters, plants and animals (Cree Nation Government 2015). However, the northern part of Eeyou Istchee has been heavily affected by industrial development and resource extraction, mainly from hydroelectric projects and mining, while much of the landscape below the northern limit of commercial forestry has already been transformed by forestry, roads and mining activity (Bélisle et al. 2021).

**Fig. 1:**
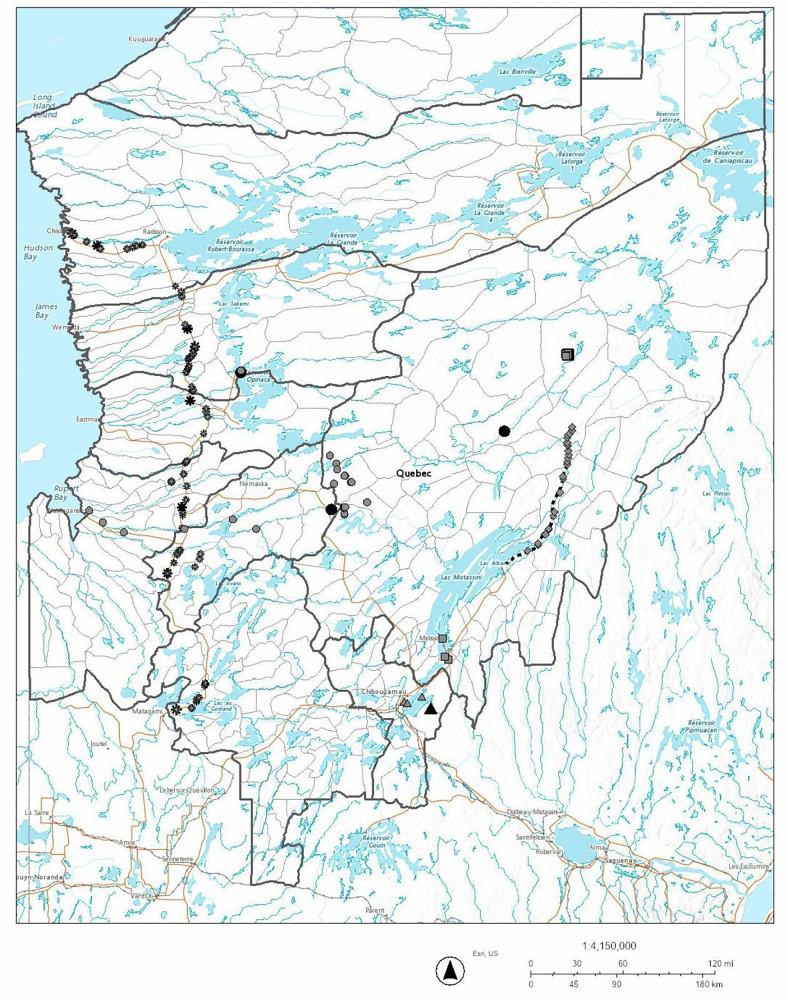
Development projects in Eeyou Istchee, and associated fish habitat compensation projects. Black symbols represent development projects, gray symbols represent the associated fish habitat compensation projects

**Fig. 2:**
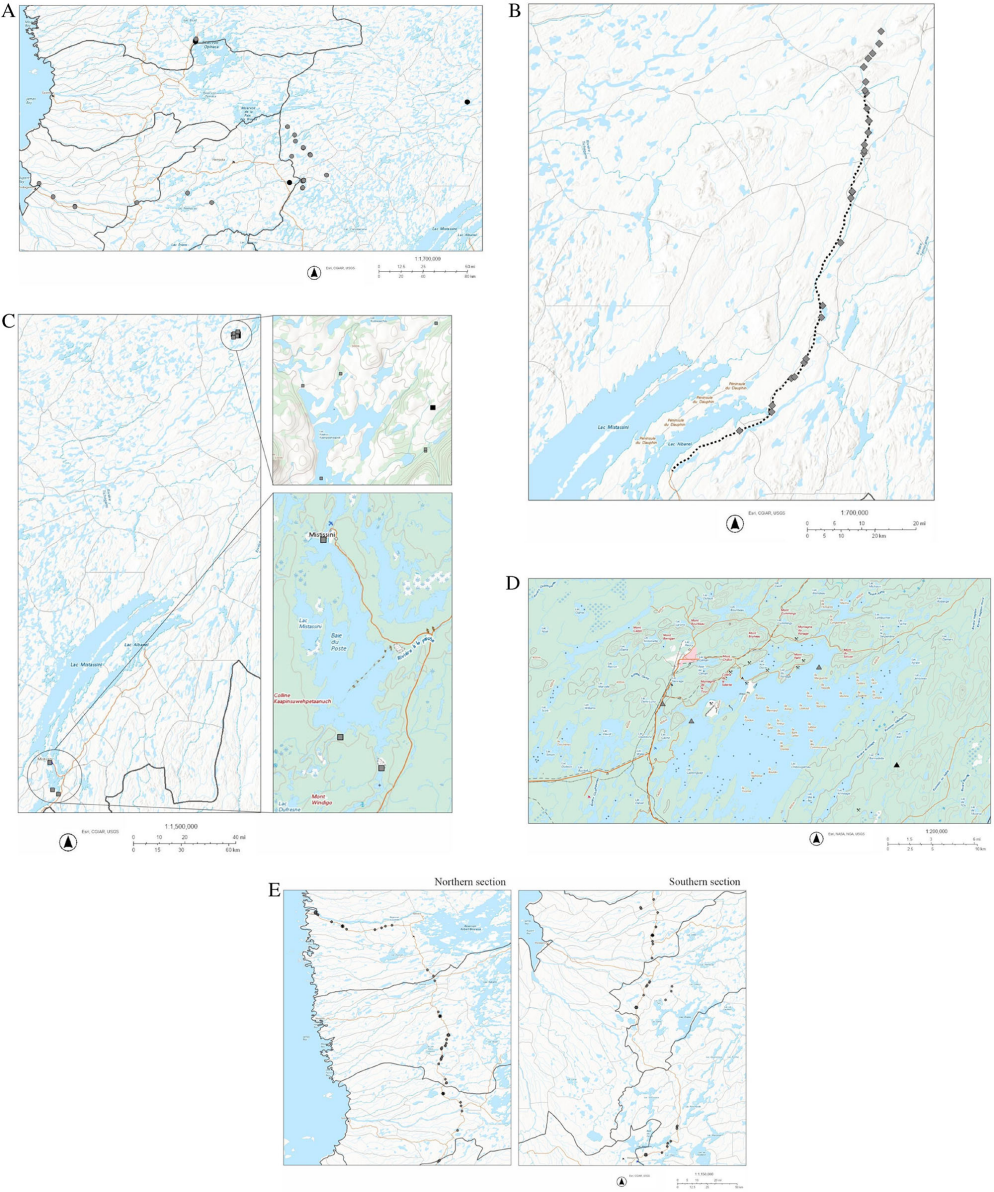
Development projects in the study area and associated studied fish habitat compensation projects. **A** Project 1: Eastmain 1A and Rupert River diversion (hydroelectric power). Black circles represent development projects, gray circles represent the associated fish habitat compensation projects. **B** Project 2: Renard Mine. Black squares represent development projects, gray squares represent the associated fish habitat compensation projects. **C** Project 3: extension of Route 167N. Dotted line represents the extension of Route 167N, gray diamonds represent the associated fish habitat compensation projects. **D** Project 4: BlackRock Mine. Black triangles represent development projects, gray triangles represent the associated fish habitat compensation projects. **E** Project 5: James Bay & Chisasibi road repair. Black asterisks represent development projects, gray asterisks represent the associated fish habitat compensation projects

In this paper, we critically evaluate the performance of fish habitat compensation in Eeyou Istchee by detailing five recent case studies (Figs. 1 and 2): offsetting the effects on fish and fish habitats due to hydropower (project 1; Fig. 2A), mining activity (projects 2 & 4; Fig. 2B, D), road extension (project 3; Fig. 2C) and road repair (project 5; Fig. 2E). The details of these recent industrial developments and their associated habitat compensation projects are summarized (Table 1), and we discuss their effectiveness, including how fish populations are affected by created spawning grounds, habitat connectivity and reconnection, and chemical contamination. The compensation projects include habitat restoration, enhancement and reconnection, fish stocking, as well as scientific monitoring of contaminants, habitat characteristics and fish populations at different sites. Information regarding Cree perspectives and knowledge were included where available. The HADDs caused by each development project, the avoidance and minimization measures taken during the compensation work, and the fish habitat compensation projects implemented to compensate for the development activities are summarized and compiled. These industrial development projects were generally constructed over a period of weeks to several years, while the compensation projects were constructed over a period of several days to weeks, then monitored for 3–5 years, sometimes 10 years, afterwards (Table 2).

**Table 1:** Summary and comparison of the five development projects and their associated fish habitat compensation projects

Project:	1. Hydropower	2. Renard mine	3. Road extension	4. BlackRock mine	5. Road repair
Type of compensation	Spawning grounds	Spawning grounds, habitat enhancement, culverts	Habitat restoration, removal of obstacles	Spawning grounds, stocking, culverts	Culverts
Number of compensation projects	Seven	Five	Two	Three	None
Inclusion of IK	Yes	Yes	No	Yes	No
Monitoring visits^a^	Annual, biannual & triannual	Annual & biannual	Annual	Annual, biannual, & quinquennial	Biannual
Size of HADDs^b^	145,700,600 m^2^	19,680 m^2^	18,050 m^2^	77,680 m^2^	6015 m^2^
Size of compensation	31,985 m^2^	3100 m^2^	4546.9 m^2^	300 m^2^	0 m^2^
Ongoing negative effects	Yes	Yes	Yes	Yes	No
Suggested improvements to increase the chances of project success	1:1 or greater compensation ratio; additional efforts (& population-level evidence) to remedy declining fish population sizes, esp. walleye	1:1 or greater compensation ratio; remediation of contamination; additional efforts (& population-level evidence) to increase brook & lake trout population sizes	1:1 or greater compensation ratio; additional efforts and extended monitoring to ensure use of spawning sites	1:1 or greater compensation ratio; more compensation to make up for lost spawning habitat due to water level changes; remediation of contamination; additional efforts (& population-level evidence) to remedy declining lake trout population sizes	1:1 or greater compensation ratio; extra compensation for the spills & sedimentation during construction

^a^Monitoring visits do not include water quality or contaminant monitoring

^b^HADDs include habitat that was destroyed, altered, disrupted, or deteriorated but do not include contaminated habitat

**Table 2:** Timelines for the five case studies of fish habitat compensation projects

Development project	Project work authorization period	Construction and operation period	Compensation authorization period	Monitoring period
**1. Eastmain 1A & Rupert River Diversion (Hydro project)**	2007–2012	2009 (transitional mode) 2012 (permanent mode)	2007–2011	2007–2023
**2. Renard Mine**	2014–2033	2014 (construction) 2033 (mine closed)	2014–2017	2015–2032
**3. Extension of Route 167 N**	2012–2016 (Lots A & B) 2013–2014 (Lots C & D)	2012–2014 (work period)	2012–2014	2015–2017
**4. BlackRock Mine**	2020–2030 (original) 2023–2033 (amended)	2023–2033	2015–2027	2016–2027
**5. James Bay & Chisasibi road repair**	2019, 2020, 2021	2019, 2020, 2021 (work period)	NA	2020–2024 (years 1 & 3 after construction)

Circles correspond to Project 1 - Eastmain 1A and Rupert River Diversion, squares to Project 2 - Renard mine, dotted black line and diamonds to Project 3 - extension of Route 167N, triangles to Project 4 - BlackRock mine, and asterisks to Project 5 - James Bay and Chisasibi road repair.

## Material and Methods

Documents related to the five case studies were the only projects that were available to be shared informally with the authors by DFO and included: 1) authorization letters (i.e., permits) from DFO granting permission for the industrial development projects, including plans for fish habitat compensation, 2) field visit reports prior to the compensation work, and 3) monitoring reports detailing the work done, and monitoring after the compensation work was completed (range: 1–10 years). Documents from both the proponent, detailing their work, and from DFO, detailing proponent compliance as well as recommendations for the compensation work, were included. Timelines for the authorization periods for the five different projects are summarized in Table 2. Unlike in Harper and Quigley (2005b), e-mail correspondence between DFO and the proponents prior to the issuance of permits, including details of avoidance and minimization measures, were not available as part of our agreement with DFO and were not included. The information that was unavailable through our informal agreement could have potentially been obtained via a formal Access to Information and Privacy (ATIP) request.

The information provided in the reports was summarized and compiled, and is presented in the following sections: 1) inclusion of Cree IK in the fish and fish habitat compensation projects, 2) authorized HADDs for each of the development projects and details of additional unmeasured impacts on fish habitats, and 3) the different fish and fish habitat compensation projects developed to offset the impacts for each of the five development projects. Additional details on the inclusion of IK for the hydropower project are detailed in Supplementary Appendix I, avoidance and minimization measures taken during the construction of the compensation projects are detailed in Supplementary Appendix II, while more details regarding the fish habitat compensation projects are provided in Supplementary Appendix III.

## Results

### Inclusion of Indigenous Knowledge

There was a wide range in how IK was incorporated, or not, into the design, implementation and monitoring of the five different fish habitat compensation projects (Table 3). IK was not included in any DFO or proponent documents for both road projects, while for the BlackRock Mine, the proponent only asked one tallyman to confirm that a potential spawning site was actually used for spawning. The Renard mine and the hydropower project both included substantially more IK, although the hydro project had far more involvement with the Crees.

**Table 3:** Inclusion of Cree knowledge in five fish habitat compensation projects in Eeyou Istchee

Project	Cree knowledge
1. Eastmain 1A & Rupert River Diversion (Hydro project)	*Extensive* Communication via committee, multiple media outlets, tallymen meetings, incorporate IK in analysis and design, Cree training & participation in monitoring
2. Renard Diamond Mine	*Moderate* Communication, helped identify compensation sites, improve project quality
3. Extension of Route 167N	*Nonexistent* None mentioned
4. BlackRock Mine	*Minimal* Tallyman identified which potential spawning grounds were used by target fish
5. James Bay & Chisasibi road repair	*Nonexistent* None mentioned

The Renard Mine mentions multiple community consultations, the sharing of environmental monitoring information to the people who used an affected trapline, the local Mistissini Band council environment manager, and a representative of the Cree Nation Government. They also considered the observations and concerns raised by the Cree community of Mistissini for their fish habitat compensation projects (summarized in Supplementary Appendix I). The hydro compensation project included greater Cree involvement and participation than all of the other projects, at all stages of project development (see Supplementary Appendix I for more details).

### HADDs

The five different development projects had a wide range of HADDs to fish habitats (Table 4). The largest HADDs, by far, were caused by the hydro project, which destroyed or permanently altered almost 150 km^2^ of fish habitat to build and operate the Eastmain-1A powerhouse, and to partially divert the Rupert River. The mines caused the next largest HADDs, although BlackRock affected about ten times the size of fish habitat than the Renard mine. Although the BlackRock mine permanently destroyed only a small area of fish habitat, substantial areas of aquatic habitat were permanently altered by the mine and contaminated by mining effluent, while the Renard mine drained at least part, sometimes all, of six different lakes. The HADDs due to the road extension were similar to the Renard mine, and also tended to have a higher portion of temporarily affected habitats due to the temporary nature of the culvert and road repair work. Although the HADDs from the road repair work were less than half of the HADDs from the road extension, when excluding the fish habitat that was affected only by BlackRock’s mining effluent, about ten times more fish habitat was permanently destroyed by the road repair work than by BlackRock Mine, a quantity of fish habitat which is not negligible.

**Table 4:** HADDs caused by the five studied development projects and associated fish habitat compensation projects in Eeyou Istchee. HADDs refer to permanently destroyed or damaged fish habitats unless specified as temporarily disrupted (i.e., disrupted)

Project	Infrastructure	Affected species	Type of habitat	HADDs	Total HADDs
1. Eastmain 1A & Rupert River Diversion (Hydro project)	5 flow release structures, 72 dikes, 8 hydroelectric structures (2 require free passage of fish), 225 km roads	Walleye, sucker, lake whitefish, lake sturgeon, lake trout, brook trout	Lakes, rivers, & streams with habitats for spawning, migration, feeding, rearing young	*Reduced flow* 16,567,000 m^2^, (516,000 m^2^ disrupted) *Diversion Bays* 230,000 m^2^ (dikes), 128,900,000 m^2^ (hydraulic changes) *Increased flow* 22,000 m^2^	145,700,600 m^2^ of fish habitat destroyed or permanently altered
2. Renard Mine	Open pits, vertical shaft, processing plant, pumping plant, processed kimberlite containment areas, explosives storage facility, housing complex, airstrip & associated facilities	Brook trout, northern pike, pearl dace, white sucker	6 lakes with habitats for feeding, rearing young, spawning	*Used for mining pit* 11,960 m^2^, 3880 m^2^ *Drained littoral zone* 730 m^2^, 2160 m^2^, 200 m^2^ *Water level changes* 80 m^2^, 670 m^2^	19,680 m^2^ of fish habitat permanently destroyed
3. Extension of Route 167N	73 culverts, 22 bridges, 13 road fills, permanent diversion of two stream sections (85 sites require free passage of fish)	Brook trout, northern pike, walleye, lake whitefish, white sucker, longnose sucker	Rivers & streams with habitats for feeding, rearing young, reproduction	*Lot A* 4970 m^2^ (180 m^2^ disrupted) *Lot B* 5780 m^2^ (180 m^2^ disrupted) *Lots C & D* 6000 m^2^ (960 m^2^ disrupted)	16,285 m^2^ of fish habitat permanently destroyed 1765 m^2^ disrupted
4. BlackRock Mine	Open pit, complex metallurgical plant, storage areas & ponds for mining waste, mining site access road, railway line extension to enable rail transportation of mining materials	Northern pike, yellow perch, white sucker, cyprinids, walleye, lake whitefish, brook trout	Lakes, rivers & streams with habitat for feeding, rearing young, migration	*Water management* 77,000 m^2^ modified *Bridge in Lake Jean* 400 m^2^ *Road repair, culvert replacement (11) & installation (4)* 280 m^2^ destroyed *Mining effluent* 192,000 m^2^ (disposal of tailings & waste)	680 m^2^ of fish habitat destroyed 77,000 m^2^ permanently modified 192,000 m^2^ contaminated
5. James Bay & Chisasibi road repair	Repair & maintenance of 112 culverts, 47 require free passage of fish	Brook trout, fivespine sticklebacks, northern pike, white sucker, longnose dace, pearl dace, spotted sculpin, walleye	Rivers and streams with habitats for feeding, rearing young, shelter	*2019* 297 m^2^ *2020* 3944 m^2^ *2021* 176 m^2^ (352 m^2^ deteriorated) *2022* 47 m^2^ (169 m^2^ deteriorated, 1030 m^2^ disturbed)	4464 m^2^ of fish habitat permanently destroyed 521 m^2^ deteriorated 1030 m^2^ disturbed

To use large construction vehicles during the compensation activities, the surrounding habitat must be sufficiently deforested to enable the machines to access the work site. This deforestation is especially extensive when no existing roads are available for use, and temporary roads have to be built. Additionally, machinery was also used to remove much of the existing stream habitat, including dirt, fine sediment, boulders and rocks, organic material and large woody debris, to create room for the new spawning ground substrate. The bottom of the streambed was also usually excavated to ensure sufficient water depth over the spawning grounds to cover any developing eggs or fry.

The sheer weight of the machinery on the banks of the waterbody also increases the likelihood of erosion or sedimentation. Proponents largely seemed to mitigate this risk through temporary stabilization structures during the work, followed by restoration and revegetation of the banks. However, for all projects, there were some compensation sites that showed some degree of insufficient plant regrowth, sedimentation or destabilization where nothing further was required. As well, water contamination through oil and gas leaks and spills still occurred despite precautions, and for one of the road repair sites that experienced a chemical spill, no further remediation was required or pursued once the original situation was addressed. In addition to reported leaks, minor unreported leaks or spills during construction are likely to have occurred.

#### Monitoring contaminants

Monitoring programs for contaminants were only included in the Renard mine compensation. Environmental discharge objectives (EDOs), the level of contamination an environment can support without altering its sustainability, were specifically established for the Renard Mine. The effluent going into Lake Lagopède will be continually monitored at three sites (i.e., 6 months beforehand – 3 years during operation), while surface water and sediment quality will be monitored during the mine’s construction, operation and closing phases. Noise pollution from explosion sites will be sampled quarterly, then yearly, during the mine’s operation (Table 2). For more details on spawning ground water quality, see Water Quality in Supplementary Appendix III.

#### Accountability

When any habitat damage occurred during construction, contractors were required to use erosion control methods. Stream banks, especially slopes, were all reforested to limit future erosion, although the type of plants was not specified and replanting native species was not required.

Proponents were required to monitor the avoidance and mitigation measures taken during the work, and to provide DFO with a detailed report, including pictures. DFO then evaluated the effectiveness and compliance of the measures used, based on how correctly the temporary (e.g., cofferdams to enable dry work) and permanent (e.g., culverts, spawning grounds) structures were installed, and how well erosion and sediment input were controlled and minimized throughout. Emergency response plans for spills or leaks, and their correct use, was also monitored by DFO. (See Supplementary Appendix II for a detailed summary of the avoidance, minimization and site remediation measures taken during the construction period of the five different compensation projects).

### Compensation

All development projects created artificial spawning grounds as habitat compensation. The hydro project created several spawning grounds for different target species, alongside additional monitoring (i.e., fish populations, environment) as compensation, while the compensation for the remaining development projects also included culvert installation or replacement alongside the created spawning grounds. The key benefit of the culverts to fish habitats was to restore the free movement of fish between water bodies, which was referred to by both DFO and proponents as a substantial benefit to fish populations. Other compensation measures included Renard Mine’s targeted habitat enhancement for walleye alongside contaminant monitoring due to this site’s location near a polluted former mine (i.e., Icon Sullivan), and BlackRock Mine’s participation in lake trout (*Salvelinus namaycush*) stocking (Table 5; see Supplementary Appendix III for a full summary of all the different compensation measures taken).

**Table 5:** Compensation done for five development projects in Eeyou Istchee

Development project	Location (# sites)	Type of compensation	Target species	Size of compensation	Total size	Goal of compensation	Unaddressed issues
1. Eastmain 1 A & Rupert River Diversion (Hydro project)	1. Fast-flowing waters on the Rupert River (4 sites) 2. Rupert River (km 290, 1 site) 3. Lemare River (tributaries & lakes of Rupert River) (7 sites) 4. Downstream release structures (4 sites, 1 site) 5. Rupert River, diversion bays (2 sites) 6. Diversion bays (3 sites) 7. Downstream of Sarcelle powerhouse (2 sites)	1. Fast-flowing spawning grounds 2. Spawning grounds 3. Spawning grounds 4. Fast-flowing spawning grounds 5. Spawning grounds 6. Spawning grounds 7. Fast-flowing spawning grounds	1. Walleye, lake whitefish, longnose sucker, white sucker 2. Lake sturgeon (mostly) 3. Brook trout 4. Walleye, suckers, & lake whitefish; walleye & lake whitefish 5. Lake sturgeon 6. Lake trout 7. Walleye, longnose sucker, white sucker, lake whitefish	1. 7096–7274 m^2^ 2. 1800 m^2^ (minimum), 3. 7740 m^2^ 4. 3085 m^2^, 72 m^2^ 5. 3900 m^2^ 6. 4157 m^2^ (minimum) 7. 11,765 m^2^ (minimum)	31,985 m^2^	Increase fish populations, collect environmental & fish population data	Declining fish populations, especially walleye
2. Renard Mine	1. Near the Renard mine (4 sites) 2. Diversion canal at Icon-Sullivan former mine (1 site) 3. Lake Lagopède 4. Near Lake Mistissini (1 site) 5. Lake Mistissini near historic spawning grounds (1 site)	1. Spawning grounds 2. Habitat enhancement 3. Spawning grounds 4. Replace two culverts, spawning grounds 5. Spawning grounds	1. Brook trout 2. Walleye 3. Lake trout 4. All fish, brook trout 5. Walleye	1. 600 m^2^ 2. 15,000 m^2^ 3. 300 m^2^ 4. 2000 m^2^ (restore access), 100 m^2^ 5. 600 m^2^	3100 m^2^, restored access to 2000 m^2^	Increase fish populations, collect data (pollution)	Water contamination (old & new mine site)
3. Extension of Route 167N	1. Lots A & B (21 sites) 2. Lots C & D (6 sites)	1. Habitat restoration, removal of obstacles (i.e., road embankments, old culverts) 2. Habitat restoration, removal of obstacles	1. All fish 2. All fish	1. 3535 m^2^ (feeding, shelter, nursery, spawning grounds) 2. 1011.9 m^2^ (feeding, shelter, rearing young, reproduction)	4546.9 m^2^	Free fish movement, increase fish populations	No signs of spawning, no more monitoring
4. BlackRock Mine	1. Lake Charley (1 culvert), Hamel Island (2 culverts) 2. Lake Chibougamau 3. Lake Chibougamau (1 site)	1. Culvert replacements 2. Lake trout stocking 3. Lake trout spawning grounds	1. All fish 2. Lake trout 3. Lake trout	1. NA 2. NA 3. 300 m^2^	300 m^2^	Free fish movement, increase fish populations	1. Loss of ~500 m^2^ spawning habitat due to water level changes, proximity to pollution 2,3. Declining Lake trout populations
5. James Bay & Chisasibi road repair	NA	NA	NA	NA	NA	Free fish movement	Spills & sedimentation during work

The main criteria used to determine the success of the habitat compensation projects was whether they remained stable over the monitoring period. The physical conditions of the culverts, including stability, were evaluated by DFO, as was the free passage of fish. Spawning grounds were evaluated based on their size and shape, and whether they were underwater for the entire spawning period. Useability was assessed via the presence of any spawners and eggs. All sites were monitored for erosion and sedimentation. However, this evaluation period was relatively short-term, ranging from 1 to 3 years for most culvert installations and spawning grounds, to over 10 years to monitor use of several of the artificial spawning grounds (See Supplementary Appendix III for details on the construction and monitoring of the different compensation projects). Long-term stability of compensation projects also includes sufficient reforestation of the surrounding habitat at the site to prevent future issues with sedimentation and erosion. In many cases, proponents that were found noncompliant if they had not implemented the measures they were asked to take which resulted in undesirable project outcomes (i.e., insufficient plant growth, erosion, sedimentation). In these cases, the proponents would be considered compliant if they made efforts to address these issues, and if they documented these efforts in their reports, even if the issues were not actually remedied. Similarly, for the lake trout stocking, it was more important for the proponent to make efforts to fulfill their stated compensation requirements, than for the compensation project to effectively increase the targeted fish populations. No additional efforts were made to increase lake trout population sizes when drastic declines of spawning stock were observed by BlackRock during the lake trout stocking program, while water contamination issues detected during monitoring (i.e., high levels of copper, aluminum) were dismissed by DFO and claimed to be due to natural causes. Although the stated objective of the Renard Mine’s compensation was to significantly increase habitat productivity and fishing success for the Crees in Mistissini, especially for brook trout (*Salvelinus fontinalis*) and lake trout, this objective was not met and no additional actions were requested by DFO when monitoring revealed that these fishing stocks were showing substantial population declines.

Similarly, monitoring for the hydro compensation projects revealed substantial decreases in fish populations, but nothing more was required by DFO. As the spawning grounds were physically intact and stable, the proponent was considered to have met their habitat compensation commitments. DFO also delayed any decisions on how to address the alarmingly low walleye recruitment observed in Rupert River in order to include an additional year of data.

No habitat compensation was explicitly required for the road repair project, as culvert replacement and maintenance was included. DFO considers the effects of culvert installation to be small, even with many culverts, as some sites required the free movement of fish. In general, DFO predicted the nearby fish habitats to be the same afterwards if the mitigation and restoration measures were properly done during the work. Despite several instances of noncompliance, including a hydrocarbon spill, fish deaths, and sediment input, no compensation was required for these unexpected HADDs. The contractors were informed of these violations until the situation was remedied, and despite being fined, DFO did not require further fish habitat compensation.

#### Monitoring

The structural integrity and stability of all of the artificially created or enhanced habitat features for compensation projects was evaluated during monitoring, as was the presence of silt in the water and the degree of erosion on the streambanks. The physical conditions of all of the culverts, including their stability, were evaluated by DFO, as was the free passage of fish. Spawning grounds were evaluated based on their size and shape, and whether they were underwater for the entire spawning period. All sites were monitored for erosion and sedimentation. For spawning grounds, the size of the submerged habitats, the water depth, and the integrity of the substrate were evaluated. The use of these compensation projects by target species for their intended purpose was verified through the presence of eggs, larvae, or spawning adults. For the road repair and culvert projects, the physical characteristics of the culverts and surrounding stream habitats, including waterflow quality, water depth and current speed, were assessed afterwards, as was the free movement of fish. Potential for upstream migration was evaluated by assessing these same characteristics as well as the height differences of the culvert thresholds. Visual assessments of fish movement were conducted during the Spring or Fall migration period. Projects were usually monitored once annually for two to three years, but spawning grounds for lake sturgeon and lake trout, included as compensation for the hydro and mining projects, were monitored for 10 years (Supplementary Appendix III).

#### Unmeasured effects

As there are no void spaces, new habitats created for compensation projects cannot be created without destroying or modifying existing habitats. The ecological impacts resulting from this destruction and disruption of the original aquatic and terrestrial habitats at the compensation sites were not considered for any of the compensation projects.

## Discussion

### Habitat Compensation: Is Trying Enough?

Overall, the priority for the five studied habitat compensation projects in Eeyou Istchee was their structural integrity and potential ability to function as designed, rather than their proven beneficial effects on fish reproduction and fish population dynamics. Even when it was clear that fish populations were declining or not recuperating despite the habitat compensation projects, such as BlackRock mine’s failed lake trout stocking efforts in Lake Lagopède, nothing further was done. BlackRock was seen as fulfilling its duty despite the declining lake trout population, as they did participate in the spawning program. Similarly, the hydro project’s negative effect on numerous populations of fish, especially walleye, was noted but no action was taken, as DFO wanted to collect another year of data, however this data, as well as any additional conservation or remediation actions taken by DFO, were not made available and are currently unknown. Similarly, complaints from Cree land users regarding the poor taste of sturgeon due to the hydro development project were noted but no action was taken. The ultimate stated goal of fish habitat compensation, to increase fish productivity at a given site to compensate for losses at another, was not explicitly evaluated for any of these projects, and no action was taken to remedy projects that were clearly failing. Consequently, for most habitat compensation projects, including these case studies in Eeyou Istchee, it is impossible to know whether or not NNL of fish productivity has even been achieved. Although these fish habitat compensation projects may be able to guarantee the possibility of fish passage through a culvert, or that a few fish have occasionally used an artificially created spawning ground, increased productivity in developed habitats or the achievement of NNL cannot be guaranteed through fish habitat compensation projects. For NNL to be achieved, there must be equivalence between what is lost and what is gained (Boileau et al. 2022; Quétier and Lavorel 2011). However, for all studied projects, the size of the HADDs due to industrial development far exceeded the cumulative size of the habitat enhancement projects, and the productivity of these fish habitats, including proxies for productivity such as population size, were not evaluated for any of the projects. As well, merely “trying” to meet standards while actually falling short of the stated thresholds is also a common practice in forestry (i.e., Teitelbaum and Wyatt 2013), but this is not enough as full compliance of proponents, and proven effectiveness of compensation projects, is not required or enforced for fish habitat compensation projects.

All habitat requirements treated HADDs as well as habitat creation in terms of size, and did not consider habitat quality in any depth, or explicitly measure fish productivity. For fish habitat compensation in Eeyou Istchee, habitat quality was only briefly addressed, except for cases when a polluted site had been chosen for habitat compensation (i.e., Renard mine’s walleye habitat enhancement). In this case, both DFO and the proponent agreed to develop walleye habitat despite pollution from the old copper mine and claimed that walleye do not usually feed on benthos. However, walleye regularly consume benthic prey sources (Pothoven et al. 2016), and walleye habitat quality, especially for spawning, is limited by the presence of metals (Bozek et al. 2011; Raabe et al. 2020). Although the consideration of both habitat quantity and quality are crucial for the rehabilitation or maintenance of fish populations (Lamothe et al. 2019; Quigley and Harper 2006a), size only measures are commonly used for fish habitat compensation in Canada (Harper and Quigley 2005a), indicating this practice has persisted despite its limits. As well, it is common for HADDs to be larger and compensation sites to be smaller in practice, than what was originally authorized (Harper and Quigley 2005b; Quigley and Harper 2006b). In several instances of the case studies, the size of the developed spawning grounds were smaller than authorized by their permit, especially due to lower water levels, but nothing further was required from DFO as the intended habitat compensation projects were completed. Sufficient data to assess habitat productivity at the development and compensation sites was also generally not available for the case studies, and thorough pre-impact assessments that enabled productivity to be evaluated were rare or non-existent, as was the use of control or reference sites in non-disturbed areas. In particular, uncontaminated reference sites would clarify baseline levels of metals for water bodies located within the Canadian shield to better distinguish between contaminants due to mining activities. A similar lack of before and after habitat data has also been observed in compensation projects elsewhere in Canada, and this situation has persisted for over two decades despite calls for improved data collection protocols (Harper and Quigley 2005b; Quigley and Harper 2006a). Repeated calls for improvements in fish habitat compensation in Canada have not been heeded, which has stalled the progress of these projects.

### Facility over Effectiveness in Habitat Compensation Projects

Fish habitat compensation projects in Eeyou Istchee were effective at addressing and minimizing bank instability, especially in terms of addressing issues with erosion or sediment input into the water. Indeed, fine sediment can destroy spawning grounds, especially when interstitial spaces between gravel or cobbles are filled with sediment and there is no room or oxygen available for fish eggs (Huang and García 2000). Fine sediment can also adhere to pelagic fish eggs and negatively impact their level of buoyancy (Corell et al. 2023), while sediment-laden aquatic ecosystems can harm fish by negatively affecting respiration, lowering growth, reducing immunity levels, interfering with migration, and impeding normal sensory, reproductive and movement behaviors (Mikołajczyk and Nawrocki 2019). However, the creation or restoration of healthy riparian zones should be prioritized over bank stability alone. Healthy riparian zones provide many benefits to fish habitats beyond erosion control (Richardson et al. 2010), including food, habitat, and shelter from predators and the physical environment (Pusey and Arthington 2003). As well, a certain level of bank erosion is a natural process in rivers and can be beneficial (e.g., Florsheim et al. 2008), while habitats with rocky riprap for riverbank stabilization tend to have less riparian vegetation and thus no stream cover, which is less beneficial for fish (Massey et al. 2017). Prioritizing native species for replanting efforts has additional benefits for conservation, as positive interactions between co-evolved native species can facilitate ecosystem restoration (Liu et al. 2014), while the replanting of native species can help to speed up the restoration of contaminated land used for mining (Gairola et al. 2023). The use of native species was also strongly preferred by Cree land users (AAM, pers. comm.).

It appears that developed spawning grounds generally tend to have positive effects on fish populations, especially for salmonid species that prefer to spawn on rocky substrates that are often limited in natural habitats (Taylor et al. 2019; Pedersen et al. 2009). However, the key factor that determines the relevance and effectiveness of a given compensation measure, is whether the project addresses a limiting factor that can have a significant effect on the fish population (i.e., Lamothe et al. 2019). Although healthy fish populations are supported by adequate fish habitats, the species-specific effects of habitat quality and quantity on fish demography are variable, and fish population declines are not always driven by a lack of quality habitat (Brown et al. 2019). In the cases that spawning habitats are limited in an environment, the creation of new spawning habitats is likely to have positive effects on fish populations (e.g., Taylor et al. 2019; Pedersen et al. 2009). However, the case studies that claimed that spawning substrates were limited used habitat assessments based only on visual observations. Potential spawning habitat was identified by visual identification, then measuring physical cues like depth, substrate and water flow. No other efforts were made to assess population dynamics or additional limiting factors. As well, spawning habitat that is actually used for spawning by target species can be difficult to accurately identify. Vast stretches of artificial spawning habitat may be considered ideal for fish spawning, yet fish will often only spawn in a small subset of the available habitat (e.g., Wyman et al. 2018). Indeed, natural factors difficult to replicate, such as under-gravel stream flow (Whitlock et al. 2014) may be crucial in the selection of natural spawning grounds. More research is needed beforehand to confirm whether spawning habitat is a limiting resource before artificial spawning grounds are created as habitat compensation projects.

Evidence of successful spawning on developed spawning grounds is an encouraging sign, but is not sufficient proof that fish habitat compensation projects are effective compensation for damaged sites. As well, habitat compensation must also support the development of juvenile fish to positively affect population-level recruitment (Knott et al. 2021). However, information is lacking on the specific habitat requirements at all life stages or typical juvenile densities for most target fish species (Smialek et al. 2019), and such insufficient knowledge may limit the success of fish compensation measures (Lamothe et al. 2019). For the case studies, information on juvenile life stages was limited to the presence/absence or quantification of eggs, larvae or juveniles for the subsequent monitoring at the developed spawning grounds. Although habitat requirements for adults, spawners and eggs were discussed for different target species, in terms of depth, substrate size and layers, the habitat or spatial requirements of juvenile fish were not mentioned. Generally speaking, the benefits of habitat compensation are often temporary and fleeting due to an overly short-term perspective (Blackmore 2020; Damiens et al. 2021). This was seen in the typical 3–5 years of monitoring or maintenance for the majority of the created spawning grounds. This limited monitoring period does not guarantee that the benefits of spawning ground development will continue in the longer term. It is possible that after several years with no monitoring, the spawning grounds could be swept away by currents, covered in periphyton, or have substantial sediment build-up (Baetz et al. 2020; Johnson et al. 2006), unless efforts are made to continually clean sediments from spawning grounds to prevent their degradation (e.g., Nagel et al. 2020). Additional maintenance measures that target sediment removal thus may be necessary to ensure the long-term benefits and viability of developed spawning ground projects (Fischer et al. 2020). However, except for the fish population monitoring for the hydro project, the purpose of the monitoring visits were to determine that the project was functional and completed as designed. These monitoring visits never assessed fish habitat productivity or population-level impacts of the compensation projects.

### Lack of Long-Term Monitoring

All of the fish habitat compensation projects in Eeyou Istchee had a short-term focus. The longest monitoring period for a compensation project was 10 years, for lake trout and lake sturgeon spawning activity in created spawning grounds, while the shortest monitoring period was only a year or two for some culverts and spawning grounds that had already met all criteria (Table 2). However, this short-term focus of habitat compensation projects can threaten biodiversity as it stands in stark contrast to the permanent loss of habitat that occurs as a result of industrial development activity (i.e., Damiens et al. 2021). At first glance, there also seems to be a large discrepancy between NNL in theory and in practice, as the habitat losses shown in the case studies seem to be quite a bit larger than the claimed habitat gains and benefits from the more site-focused habitat compensation projects. For example, in comparing habitat losses and HADDs for each industrial development project and the association compensation with habitat gained through enhancement and reconnection, the achievement of NNL of fish habitat probability between the two seems impossible. This problem may be systematic, leading to current regulations and administrative practices that are authorizing a Net Loss of fish habitat productivity across Canada (Favaro and Olszynski 2017). Generally speaking, habitat compensation seems to be more effective at slowing, rather than stopping, environmental destruction caused by industrial development (Damiens et al. 2021). This may be due to the general “*yes, and…*” approach to granting permits for industrial development that tends to avoid outright refusal (i.e., Bull et al. 2022; Phalan et al. 2018; Teitelbaum and Wyatt 2013).

### The Value of Including IK in Compensation Projects

Three of the five projects included IK, with HADDs that were 1.2–10,000 times larger than the two projects that did not include IK. For example, the hydro project had substantially larger HADDs relative to the other projects, while also including substantially more IK in the compensation projects and their monitoring. The projects with the smallest HADDs, the road repair and road extension, used no IK in the project. Both mining projects used some IK, however the Renard mine permanently destroyed a greater quantity of fish habitat, and also used substantially more IK relative to the BlackRock mine. This variability reflects the scope of consultations with Indigenous communities required of proponents, whereby projects with a high risk of causing irreplaceable HADDs (i.e., hydro, mining) have more obligations to the communities, while lower risk projects (i.e., road repair & road extension) do less within the scope of required consultations (Brideau 2019; Wright 2020). Similarly, the extent of prior impact assessments was also positively related to the size of the HADDs caused by the developments. For example, the most comprehensive prior impact assessment was undertaken for the hydro project, followed by the mines, then the road extension, while the documents for the road repair work did not mention any prior impact assessments.

Both mining compensation projects addressed water contamination issues by including water quality monitoring within their compensation projects. However, when elevated water quality measures were detected, they were dismissed as naturally occurring due to the geological composition of the Canadian Shield, which has naturally high levels of numerous substances. Although many chemicals may occur naturally, high levels of anthropogenic pollutants are added to aquatic ecosystems through industrial activities (e.g., Carignan et al. 2003), and no efforts were made to distinguish between the two sources. For these compensation projects, the effects of mining (e.g., former copper mine, current mine) were monitored yet unaddressed. Under the Fisheries Act, proponents must agree not to discharge harmful substances unless otherwise authorized (DFO Fisheries and Oceans Canada 2019), but authorized effluent release does not require remediation or compensation. Similar to the chemical contamination of aquatic habitats in Eeyou Istchee, water contamination issues are widespread in Indigenous communities in Canada (Bradford et al. 2016; Sarkar et al. 2015) and worldwide (e.g., Yazzie and Baldy 2018). Mining practices in particular threaten food security and sovereignty for Indigenous peoples and local communities around the world due to chemical contamination (Blanco et al. 2023). In many Indigenous communities, including in Eeyou Istchee, technical top-down water management practices are preferred over management using IK (Baijius and Patrick 2019). However, future fish habitat compensation projects in Eeyou Istchee may also benefit from the inclusion of Cree perspectives and knowledge for any water issues likely to affect the Crees within any agreements with industry or DFO, like habitat compensation projects in Australia (e.g. Jackson and Barber 2015).

Although the mining projects monitored water contamination at the compensation sites, remediation of the chemical contamination was not considered for the fish habitat compensation. Future inclusion of water contamination into the monitoring of fish habitat compensation projects in Canada could be facilitated through the use of the Habitat-Based Resource Equivalency Method (HaBREM), which determines the degree of compensation required to restore the damage by modeling sublethal injuries in multiple indicator species within a given habitat (Baker et al. 2020), or by accounting for the extent of resource loss experienced by Cree land users following industrial activity (e.g., Duffield et al. 2021). However, it can be difficult to assign responsibility for who will clean up water contamination, especially for legacy contaminants or when companies are no longer in business. Future fish habitat compensation projects would also benefit by taking more of an ecosystem approach that considers the cumulative effects of development within a region. This includes projects meant to restore connectivity through the removal of dams (Tang et al. 2021) and road-crossing barriers (Diebel et al. 2015), as well as process-based restoration of rivers and streams, whereby infrastructure is removed to enable the resumption of natural ecosystem processes (Ciotti et al. 2021). Fish habitat compensation projects on a larger scale or on a longer-term timescale may be more likely to better balance the HADDs caused by industrial development and may thus increase the likelihood of achieving NNL (e.g. Favaro and Olszynski 2017).

## Conclusions

For future fish habitat compensation projects in Eeyou Istchee, holding proponents accountable to the consequences and effectiveness of their fish habitat compensation projects, rather than just the completion of the planned work, will also help to ensure that all fish habitat compensation projects have positive impacts on fish and fish habitats. Indeed, trying to address these issues through fish habitat compensation projects is not enough, and there is a crucial need for scientists to develop monitoring and assessment frameworks that policymakers could then use to improve regulations for industrial development projects. Focusing on the full effects of the development project on fish and fish habitats could also increase compensation for any additional losses that are not usually accounted or compensated for (Moreno-Mateos et al. 2015), such as those due to repeated habitat disturbance during the construction phase (Haghkerdar et al. 2019) or issues due to noncompliance. As well, the loss of natural resources experienced by Indigenous communities, especially losses driven by water contamination, should also be accounted for and compensated (e.g., Duffield et al. 2021). As the outcome of fish habitat compensation projects are highly uncertain, with many different factors that can affect it (Moilanen et al. 2009; Weissgerber et al. 2019), projects with a greater compensation ratio (Minns 2006; Minns and Moore 2003), as well as larger sized projects (Greig et al. 2022), tend to be more successful in the face of uncertainty. For future fish habitat compensation projects, it is recommended that they at least match the size of the HADDs, rather than being smaller, and that the quality and effectiveness criteria of the compensation project is met. Taking these measures will increase the chances that each individual project can achieve NNL, as well as increasing the likelihood that some individual projects will overperform, enabling NNL to still be achieved overall even if some projects underperform. Finally, fish habitat compensation projects in Eeyou Istchee may benefit from increased inclusion of Cree knowledge, especially at earlier stages of project development (i.e., site selection for compensation, identification of conservation needs), as well as including the perspectives and expertise of Cree people when evaluating project outcomes. Due to limited access to information, we were only able to study five cases in Eeyou Istchee. Nevertheless, our in-depth analysis allowed us to highlight several common themes that suggest wide-ranging issues. However, in order to evaluate how common and widespread those issues are across Canada, additional studies in comparable environments would be needed.

## Supplementary information

Supplementary material

## Data Availability

No datasets were generated or analysed during the current study.
