# Supplementary material for: Case Studies of Fish Habitat Compensation in Eeyou Istchee: Compensation Projects Prioritize Facility over Effectiveness: Fish Habitat Compensation in Eeyou Istchee: Is Trying Enough?
Source: Environ Manage. 2025 Sep 15;75(12):3384–400. doi: 10.1007/s00267-025-02276-1 (PMC12575471; doi:10.1007/s00267-025-02276-1)
Supplement: Supplementary file 1 — Supplementary material [file 267_2025_2276_MOESM1_ESM.docx]

**Supplementary material for**

**Case studies of fish habitat compensation in Eeyou Istchee:**

**compensation projects prioritize facility over effectiveness**

**(working title: Fish habitat compensation in Eeyou Istchee)**

Kathleen Church^1*^, Adriana Raquel Aguilar-Melo^1^, Hugo Asselin^3^, and Katrine Turgeon^1^

^1^Université du Québec en Outaouais, ^2^Université du Québec en Abitibi-Témiscamingue

*corresponding author, email: [kathleen.church@mail.mcgill.ca](mailto:kathleen.church@mail.mcgill.ca), ORCID:0000-0002-7096-6869

**Appendix I**

***Inclusion of IK in Fish Habitat Compensation***

For the Renard mine fish habitat compensation, a tallyman also provided IK which helped confirm if the sites chosen for walleye (*Sander vitreus*) habitat enhancement and spawning were used by walleye. The advice of a tallyman was also helpful to improve the quality of the created walleye habitat, as the tallyman mentioned that there were boulders with sharp edges that could harm the walleye and needed to be removed or replaced. A Cree community member was also on-site during the construction of the walleye spawning grounds. The spawning ground monitoring reports mention that local communities claim lake sturgeon (*Acipenser fulvescens*) in the Rupert River upstream migration channels do not taste as good and should not be eaten. They also noted where lake sturgeon were more or less abundant than in previous years, and claimed that fish from a particular site were less fatty and had whiter flesh.

To facilitate communication and disseminate information between the proponent (i.e., Hydro Quebec) and the Cree communities, a monitoring committee was formed for the hydropower compensation. This committee included community members representing each of the six Cree communities affected by the hydropower project (i.e., Mistissini, Nemaska, Waskaganish, Eastmain, Wemindji, Chisasibi), as well as representatives from Hydro-Quebec and Niskamoon corporation, a Cree company that facilitates interactions between the Crees and Hydro Quebec. Information was also shared during two meetings with tallymen and the proponent: tallymen learned about the activities planned on their land for the following year during the winter, and heard the previous years monitoring reports during the summer. Information was also provided to the communities via a range of media targeted towards the Crees, including monthly radio broadcasts, printed advertisements, and an informative website with video capsules where Cree workers, tallymen, and community representatives shared their experiences with environmental monitoring activities for the project (website: http://www.hydroquebec.com/hydloandfriends/videos.html). Additional communication activities directed toward Cree community members were also conducted in Abitibi-Temiscamingue as part of “The North and its Logistics” conference, which was organized by the Secretariat to the Cree Nation Abitibi-Temiscamingue Economic Alliance.

Substantial IK was applied to the hydro project compensation project, at the communities’ suggestion. For example, Cree knowledge regarding ice and ice cover was included in the data analysis and interpretation, such as the mechanical properties of black ice and how the slush layer is used as a source of drinking water for the Crees. IK also provided information on the spawning and overwinter habitat use for the anadromous cisco populations (*Coregonus artedi*) present in the Rupert River and its main tributaries, which informed on the best time periods, sampling stations and fishing gear to use for the monitoring of these populations. IK was also combined with SK to demonstrate that the Nottaway river ciscos, long considered by the Crees to be a different species due to their greenish black colouration, small eggs, and different sized bones, were a genetically and morphologically distinct species, relative to ciscos in the Rupert and Broadback rivers.

Cree community members from the communities and traplines affected by the hydro project participated in the field surveys, as well as the monitoring program for the hydro project. As part of the monitoring program, Crees were hired and trained on how to sort and count cisco larvae captured by nets, and were involved in the monitoring of the riparian and aquatic vegetation found in and around the Rupert River diversion bay. Additionally, programs were established in Nemaska and Waskaganish to facilitate Cree fishers to voluntarily share the number of lake sturgeon and cisco they captured, as well as the location and gear used. IK from local community members in Nemaska was also considered in the lake sturgeon monitoring program, as they confirmed to DFO and to Hydro-Quebec that lake sturgeon do not use two of the three newly created spawning grounds, and that these spawning grounds provide nothing to the community (i.e., Rupert river km 333, Misticawissich River km 30.5).

**Appendix II**

***Avoidance and Minimization (including on-site remediation)***

*Erosion and Sedimentation*

Most of the avoidance and minimization measures focus primarily on avoiding erosion of the banks and sediment input into the water, and were similar across all compensation projects. For the culverts, erosion was limited by minimizing deforestation of the surrounding area and protecting the natural banks through temporary stabilization structures during the work, such as geotextile membrane or straw mulch. By ensuring that these temporary structures were stable, even in the case of a flood, sedimentation was also reduced. As well, further sedimentation during the work was prevented by avoiding the discharge of any debris into the water, by installing sediment barriers around the work site before the construction began, and by placing temporary sediment traps or filter berms in ditches adjacent to the work sites. These temporary structures were removed once sufficient vegetation regrowth occurred, but in some cases, permanent filtering berms were installed. The banks were stabilized and seeded for restoration. No prevention or minimization measures were taken to avoid beavers blocking the culverts (*sensu* Asselin 2015).

The Renard mine project made efforts to minimize the presence of fine sediment, especially on or nearby any known spawning grounds. Sedimentation within the created diversion canal was minimized by stabilizing the streambed and using non-absorbent substrate to reduce water loss. Additional efforts to minimize erosion and sedimentation during the work period included keeping the water pumps on a float to reduce bank erosion and using a flood-resistant large-capacity sediment pond to hold the pumped water. Ditches were created to direct water flow away from water bodies and towards vegetation to filter out sediment, and temporary structures such as berms, sediment barriers, sediment retention ponds and sediment traps were used to catch sediments during the work. During rainy weather, any excavation work or moving of materials was cancelled and sediment control practices were used at work sites. Erosion in disturbed habitats was minimized through the use of riprap and geotextile to stabilize banks during the work, and through hydroseeding and stabilization following the work. For the spawning grounds, deforestation was minimized by using existing access roads, sediment input was minimized by using turbidity curtains during the work, and erosion was reduced by restoring the banks near the spawning grounds via revegetation with native plant species after the work. For the hydro spawning grounds, geotextile membrane was used to reduce sedimentation. Erosion was prevented by using rockfill stabilization on the streambank, as well as by placing a barrier made of concrete blocks between the spawning ground and the banks of the stream; this concrete block barrier also served to increase the long-term stability of the habitat development.

*Working Dry*

Efforts to create a dry work site were motivated by reduced sediment input into the water. All of the created spawning grounds and culverts were installed using a dry work site, and cofferdams were used during the installation of virtually all of the created spawning grounds. For the road repair work, incoming stream water was blocked using either steel plates, an aquadam, pumping wells, or a fine particle cofferdam covered with geotextile. Stream water was then pumped into vegetation at least 30 metres from the stream to filter suspended sediments. A pump was used to maintain downstream flow during the work through upstream-downstream pumping. To minimize sediment input and pollution and keep the stream water out of the work sites, BlackRock Mine used temporary dams, or dikes and cofferdams (i.e., concrete sills), while the Renard Mine and the hydropower compensation projects used cofferdams whenever possible.

*Spawning Ground Substrate*

The use of the correct size and shape of spawning substrate for the target species was prioritized by virtually all spawning grounds. As well, efforts were also made to ensure that the substrate was washed and sediment-free to keep fine sediment out of the spawning grounds. For the hydro project, the substrate was filtered through a 400 mm grid, BlackRock Mines ensured that the substrate materials they used were cleaned and sediment-free, while the Renard mine ensured the stones they used for substrate were dusted beforehand to remove fine particles.

*Timing*

Virtually all compensation projects timed their work during low-water periods when the chances of sedimentation input into the water during the work are lower. They also avoided sensitive ecological periods, including spawning or migration periods for particular fish species.

Road extension work was completed outside of the spawning periods of Fall (e.g., brook trout, *Salvelinus fontinalis*; lake trout, *Salvelinus namaycush*; lake whitefish, *Coregonus clupeaformis*) and Spring (e.g., walleye, *Sander vitreus*) spawning fish. In-water work was also forbidden during the walleye upstream migration period. Culverts for the road repair work were removed and replaced during a time period with a low risk of negatively affecting brook trout (e.g., June 1st to September 15). Construction work for BlackRock Mine was timed to protect Spring spawning sites (i.e., July 15 – April 15), to protect salmonid spawning sites (i.e., July 15 - August 31), and to reduce erosion and sedimentation by working during the low-water period (i.e., July 15 to September 15). For the Renard mine, construction work was also done during the low water period, while the diversion of rivers and streams, the drainage of lakes, and culvert installation was done outside the brook trout spawning period (i.e., September 1 to November 1), while work on Lake Lagopède was completed between May 30 to August 31, to avoid negative impacts on both Spring and Fall spawning fish. The timing periods for the work periods of the hydro project or their compensation projects were not mentioned in the associated documents.

*Circulation of Fish During Work*

Temporary diversion canals, with sufficient water depth to ensure normal habitat functions, were used during the work period to maintain the free circulation of fish during the road repair work, and during work on the Renard mine for habitats downstream of the work site. Construction work was timed to be outside fish migration and spawning periods (see Timing).

*Fish Mortality*

Efforts were made to use filters on water intake pumps to avoid fish aspiration for the road repair and BlackRock mine. BlackRock mine also made efforts to catch and relocate any visible trapped fish when draining a work area or waterbody. The hydro project made efforts to capture and relocate trapped fish during dramatic water level changes from the temporary diversion of the Sarcelle powerhouse for construction of the new hydropower project. Both mining projects used fish barriers to prevent fish from migrating into any areas to be drained. For the temporary diversion of the Sarcelle powerhouse during the construction of the new hydropower project, trapped fish were captured and relocated as water levels became lower.

*Water Flow Levels*

For the culvert compensation project with BlackRock Mine, efforts were made to ensure water flow through the culverts even in low flow conditions. To do this, they used large stones to create flow pathways within the culverts, which they embedded into the substrate using a shovel. For the hydro project, two permanent upstream migration channels in the Rupert River enable fish passage for numerous fish species located downstream of the hydropower infrastructure.

An instream flow regime was implemented in the Rupert River to reduce disruptions from the hydropower project on fish populations. Designed to align with the natural hydrological cycle, the regime enables natural annual spawning cycles: spring spawning, transition, summer feeding, transition, fall spawning, transition, incubation of lake whitefish eggs, and transition.

*Additional Measures to Reduce Habitat Damage During Work*

Additional measures to reduce environmental impacts were mentioned by both mines. BlackRock Mine avoided the use of explosives in or near any fish habitats, while the Renard mine verified through their environmental consultants and DFO’s records for Eeyou Istchee that no SARA species were detected in or near any of the designated work sites.

*Contamination*

Efforts were made to minimize chemical contamination during the work periods. Contaminants and spills from construction machinery and generators used for the road repair work were minimized by placing a waterproof container under any machinery less than 20 m from the water, while spill kits, targeted for hydrocarbons, were on site for almost all sites. For the BlackRock Mine compensation projects, spills and contamination were prevented by keeping all equipment on land, doing maintenance and fueling far from the water, using clean leak-free machines that used vegetable hydraulic oil, and by removing unneeded machinery after use. An emergency spill plan was always available, as were emergency equipment and trained staff.

To ensure water quality standards were met, the Renard mine constructed a permanent mine wastewater treatment plant and a diffuser at the mine effluent outflow. A diffuser is a mechanism that releases the mining effluent into the main current of the receiving waterbody to increase dilution by facilitating mixing and dispersion between the effluent and waterbody. They also continuously monitored all quarries, sand pits and waste disposal sites located within 20 m of the high-water mark, to ensure no sediment input or altered drainage patterns occurred.

**Appendix III**

**Details of Compensation Projects**

*Monitoring*

For the hydro project, thermal stratification, a requirement for optimal lake trout (*Salvelinus namaycush*) habitat, was observed for 3 years after the creation of the diversion bays. The use of the brook trout (*Salvelinus fontinalis*) and lake trout spawning grounds outside the diversion bays were monitored for 5 years (i.e., years 1, 3, 5), while BlackRock Mine’s walleye spawning ground was monitored for 10 years after its creation (i.e., years 1, 3, 5, 10). The lake sturgeon (*Acipenser fulvescens*) and lake trout spawning grounds within the diversion canals were monitored for 10 years after the first detected use (i.e., years 1, 3, 5, 7, 10).

*Multi-Species Spawning Grounds*

Multi-species spawning habitats targeting walleye, lake whitefish, longnose sucker (*Catostomus catostomus*) and white sucker (*Catostomus commersonii*) were developed in fast-flowing waters to compensate for the hydro development. Monitoring revealed unsuitable jagged-edged blasting waste rocks were used as substrate for the spawning grounds downstream of the Sarcelle powerhouse. Substantial silting was observed, even after sand was removed via sieving and two upstream groynes were constructed. Nothing further was done, as water flow patterns were expected to improve after the commissioning of the Sarcelle powerhouse. Despite this, walleye, longnose and white sucker, and lake whitefish eggs were captured (via drift nets).

*Walleye Spawning Grounds*

A historic walleye spawning ground in the upstream channel of Lake Mistissini was destroyed by a sawmill in the 1960s, and BlackRock Mine developed a new spawning ground close to the original site. Monitoring revealed the spawning grounds were not submerged and no fish (via non-lethal fishing) or eggs (via ovocatchers) were captured on or near the spawning grounds for two subsequent years after their creation. The proponent was required to lower the depth of the spawning grounds to ensure sufficient water cover during the full spawning and incubation period, followed by more monitoring and temperature measures at this site to precisely determine when the walleye spawning period begins and ends (e.g., 5.6 ˚C until 11 ˚C).

*Lake Sturgeon Spawning Grounds*

For the hydro project, multi-species spawning habitat mainly targeting lake sturgeon was developed on the Rupert River, while spawning grounds targeting lake sturgeon, walleye and lake whitefish were developed on the Eastmain River. Monitoring revealed these new spawning grounds were used for spawning by walleye, longnose and white suckers, cisco (*Coregonus artedi*), lake whitefish, and lake sturgeon.

The hydro project’s impoundment of the diversion bays caused the loss of two confirmed lake sturgeon spawning grounds in the forebay. New spawning grounds were created prior to the creation of the diversion bays, located near the former spawning grounds but outside the hydro project’s zone of influence. However, less than half of the created spawning grounds were suitable for lake sturgeon spawning due to poor hydrological conditions, such as being too slow and deep (e.g. Misticawissich River). Consequently, these spawning grounds were not used.

*Lake Trout Spawning Grounds*

Lake trout spawning habitats were built in the diversion bay to compensate for the hydro project, but none were in ideal habitats for Lake trout, as the temperature was too high and the dissolved oxygen too low. The habitat with the best conditions (e.g., Cabot Lake) lacked thermal stratification, a key lake trout habitat requirement. However, these sites were deemed adequate for adult lake trout as telemetry revealed that they were being used by lake trout in the summer.

DFO claimed the strong homing behaviour of lake trout is likely behind their lack of use of the new spawning grounds, but they are also using the natural spawning grounds less as the habitat quality of the natural spawning sites has declined after the diversion of the Rupert River. Marked increases in depth (e.g., 1 m to 2-4+ m) and the increased presence of organic matter (e.g., periphyton, large woody debris) has made some sites completely unusable. Although no lake trout spawners were detected, one egg was found at a new spawning ground in Cabot Lake. Although no Lake trout eDNA was found in Cabot Lake, the discovery of the egg marks the beginning of a 10-year monitoring period of Lake trout spawning. However, since Cabot Lake has very poor dissolved oxygen conditions (2015: hypolimnion DO 1.1 mg/L, lake trout usually require DO 5-6 mg/L), temperature and dissolved oxygen will also be monitored for 10 years.

The Renard Mine expanded an existing lake trout spawning ground in Lake Lagopède. Water quality was also monitored at this site (see below). No eggs were detected (via ovocatchers) and no lake trout were observed (via cameras) during the first year of monitoring. However, the use of these expanded spawning grounds by Lake trout was confirmed two years after its creation after both male and female spawners were captured (via non-lethal fishing).

BlackRock Mine developed lake trout spawning grounds in Lake Chibougamau at a carefully chosen site (e.g., near a deep pit, slope >20%, bank depth < 2 m). The spawning grounds were deemed effective as the interstitial spaces were abundant and the right size (range: 15->70 cm), and the mean depth was 2.3 m. Fish, but no lake trout, were observed at this site.

*Brook Trout Spawning Grounds*

The hydro project developed brook trout spawning habitat in the tributaries and lakes of the Rupert River. Substrate was placed parallel to the current to isolate the spawning grounds, and boulders were placed nearby to slow the water current and to provide shelter for smaller fish.

The Renard Mine developed sill-pit-spawning grounds for brook trout near their mine on Lake Lagopède to increase spawning habitat availability, and removed obstacles (i.e., small islands) to upstream migration. Although a population of brook trout are found in Lake Lagopède, there are only a few brook trout spawning grounds in this lake. For sill-pit-spawning grounds, riprap weirs were built, then gravel was placed in the small pools created by the sills to create a spawning area. Gravel boxes were also placed at locations where low quantities of gravel appeared to limit brook trout spawning. During monitoring, brook trout were detected (via electrofishing), as were spawning brook trout and fry (via visual observation), confirming the use of these habitats for brook trout reproduction. All habitat developments allowed the free passage of fish, and no erosion was found on the stream banks. Physico-chemical measures of water quality were generally adequate, although pH was lower for sites closer to the Renard mine, and some sites had pH levels lower than 4, which may impede egg and fry survival.

Sill-pit-spawning grounds were also created by the Renard mine near the two replaced culverts near Lake Mistissini. Sills were created upstream and downstream of the culverts to raise the water level, reduce the current speed, and increase fish habitat; stones were also placed near the sills to slow the local streamflow and provide shelter. Although an insufficient quantity of spawning habitat was created at this site, plans to develop additional spawning grounds were not pursued as high quality brook trout habitat was detected nearby. Fish could pass freely during monitoring, and adult and juvenile brook trout were detected on one site, although no spawning brook trout were detected near the spawning grounds (via observation, electrofishing). The site’s physico-chemical values were suitable for egg incubation and juvenile development.

*Water Quality*

The Renard mine evaluated water quality and suspended solids from mining effluent in Lake Lagopède to ensure they would not impede egg and fry survival of lake trout at the new spawning grounds. Water quality was monitored before and after spawning, and under the ice in winter, every year for 3 years, then every other year until 17 years afterwards. At least three samples were taken near the substrate in the spawning grounds to measure turbidity, pH, temperature, suspended solids, conductivity, total phosphorus, fluorides, metals (Al, Br, Ur & Rd), and ammoniacal nitrogen. Mine effluent and sand or silt accumulation within the spawning grounds was monitored for three consecutive years. In 2017, three water quality values were above the standards for surface water quality: aluminum, the biochemical oxygen demand for five days (BOD5), and petroleum hydrocarbons (C10-C50). In 2018, the BOD5 was more than twice as high as the value for protection of aquatic life, aluminum again exceeded the standards for surface water quality, but only traces of petroleum hydrocarbons were detected. As aluminum levels were said to be naturally higher in water bodies on the Canadian shield, the MDDEFP was referenced to claim that high aluminum is not a concern for water with a pH between 6.5 and 9 with hardness > 10 mg/L. The overall physico-chemical properties in Lake Lagopède were deemed adequate for lake trout, including the survival of eggs and fry.

The physico-chemical characteristics of the Renard Mine’s new walleye habitat enhancement at the Icon-Sullivan site were evaluated in 2017 to establish a baseline reference. At least three sites were sampled in both spring and summer, with 5 consecutive years of follow-up. Water quality was evaluated in spring and summer, while sediments were only sampled in the summer. Measurements included pH, conductivity, metals (Al, Sb, Ag, As, Ba, Cd, Cr, Co, Cu, Mn, Mo, Ni, Pb, Se, Na, Zn), PAH, HCP F1 to F4, turbidity, temperature, suspended solids, and conductivity of the surface waters, as well as particle size and TOC in the sediments. In 2017, aluminum, zinc, and copper concentrations were the highest, although barium, calcium, magnesium, manganese, nickel and sodium were also detected. Measures exceeded threshold values for the protection of aquatic life for aluminum and copper at some sites. Although aluminum exceeded the guidelines for the protection of aquatic life at three sampling stations, no action was taken. This is because the bioavailable fraction of aluminum met the criterion for chronic effect (i.e., 0.087 mg/L), while the bioavailable fraction of metals was said to be low for fish (Roche 2012). Copper concentrations in spring also exceeded the criteria for the protection of aquatic life at two sampling stations. However, aluminum and copper were said to be naturally present in the Canadian shield, with high copper levels likely due to natural geology and not the mine. As well, since the copper was mostly in the benthos, it was determined to not pose a significant threat to walleye as walleye were said not to usually consume food from the benthos. As the water quality generally fell within guidelines, walleye development and reproduction was determined to not be constrained due to water quality issues.

*Lake Trout Stocking*

The province of Quebec began stocking lake trout into Lake Chibougamau in 2009, and BlackRock Metals agreed to participate in the ongoing stocking program (i.e., in the years 2015, 2017, 2019, 2021). Potential spawning grounds in Lake Chibougamau were first confirmed by the capture of spawning lake trout, then later used to capture the spawners used for artificial fertilization. Similar numbers were captured before and after BlackRock’s participation (Supplementary Table X.4).

Originally, the plan was to stock 20 000 to 30 000 juvenile lake trout (~1.5 years old, 12-15 cm length) into pits in Lake Chibougamau prior to thermal stratification, then monitor survival two years later (via non-lethal fishing). To identify stocked fish, the adipose fin was removed prior to release. Assuming a survival rate of 50-60%, between 40 000 to 60 000 eggs would be collected from Lake Chibougamau spawning grounds, then transported to fish farms to be fertilized and raised. However, an insufficient number of lake trout eggs were collected.

In the first year, the female lake trout spawners were small and produced only a few eggs (Supplementary Table X.2). When transported to the fish farm, over 1/3 of the eggs were dead upon arrival, and a majority of the remaining eggs died over the next month. Even fewer eggs were harvested the next time, and these were deposited into Lake Chibougamau spawning grounds rather than transported to the fish farm. The situation continued to worsen, and the next year an insufficient number of spawners were captured in Lake Chibougamau, so artificial spawning was done in Lake Dorés and the fertilized eggs were placed on the newly created Lake Chibougamau spawning grounds. DFO mentioned that the reasons for the declines in lake trout spawning stock were unknown, but that they mirror the declines observed in fishing success.

*Telemetry*

For the hydro project, the use of the new spawning grounds by lake sturgeon and lake trout were monitored using telemetry. In 2009, 50 lake sturgeons were captured and tagged and 25 receivers were placed in the diversion bay in 2011, with the exits closely monitored. Some spawning activity was suspected in 2011 at the natural spawning grounds, but there were too few tagged fish to assess spawning activity in 2012. There was no evidence that the lake sturgeons used the new spawning grounds in 2015. DFO claimed this was most likely due to their strong homing instincts and stronger preference for their natural spawning grounds, and that this would improve over time; no evidence was provided to support this prediction and no further actions were taken. In 2011, 40 lake trout were captured and tagged. Spawning activity was suspected but not confirmed in 2011, due to an aggregation of lake trout near the new spawning sites for a few days. No tagged fish were detected near the new spawning grounds in 2012 or 2013, no eggs were found, and no spawners were seen by divers in 2010. Seven new receivers were placed near the new spawning grounds in 2013, but no signs of spawning were detected.

*Walleye Habitat Enhancement*

An existing diversion channel at the Icon-Sullivan former mine site was enhanced by the Renard Mine to improve habitat quality for walleye (Figure 2B). Spawning grounds, groynes and shelters were created within ~15 000 m^2^ of the canal for walleye breeding and shelter. As this site is polluted, water quality of the sediments and surface were continually assessed (see above).

*Additional Monitoring*

Several fish populations were monitored for the hydro compensation projects, including the spawning habitats of anadromous lake cisco on the Rupert River and the population dynamics of fish communities in the Rupert River and in the diversion bays. Fishery yields and population dynamics were monitored in years 2, 7 and 12 after the Rupert River diversion, while walleye yields and population dynamics were monitored in years 2, 5, 7, 9, 12 and 14 after the impoundment of the Rupert diversion bays. The number and weight of the captured fish, their growth and condition factors, and the approximate size and age distribution of the population were assessed for each target species. The relative abundances of the different species was also evaluated. Sampling occurred over multiple years to account for interannual variation.

Additional monitoring was also conducted for the thermal regime downstream of the hydro release structures, the riparian and aquatic vegetation downstream of the hydro release structures and in the Rupert River estuary, the ice regime and presence of frazil ice (i.e., loose, randomly oriented needle-like ice crystals that sink) on the Rupert River, the saltwater intrusion in the Rupert River Bay and in the mouth of the Pontax river, and total organic carbon (TOC) in the Rupert River estuary, which affects fish growth and may be affected by reduced flow rates.

Fish habitat losses on the Eastmain River were monitored after the commissioning of the Eastmain-1 powerhouse, to obtain baseline data to evaluate its ongoing impacts. No science based assessments were made of the habitats or the fish species present prior to their destruction.

*Culverts*

The Renard Mine replaced two existing culverts in a tributary of Lake Mistissini to restore access to ~2 000 m^2^ of previously inaccessible upstream habitat. Riprap sills constructed for brook trout spawning also raised the water levels to ensure water backflow in the culverts.

The road extension project included the replacement of ineffective culverts, the addition of a bridge, and the removal of backfill or road embankments that impeded fish movement. Vegetation regrowth was insufficient at the first monitoring, but had improved at the second monitoring, when all sites were found to be stable and to allow free movement of fish. After the compensation sites were found to be sufficient at the second monitoring, further monitoring was not deemed useful as these sites were not expected to deteriorate over time, and was cancelled.

BlackRock Mine replaced culverts at two sites: a culvert blocked by a beaver dam at Lake Charley, and two culverts at Hamel Island, where one culvert is above the water line while the other is clogged with sediment. The Lake Charley culvert will relink Lake Charley with Frontiersman Lake, which the proponents claim will improve ~2500 m^2^ of fish habitat upstream, downstream, and within the culvert (unverified estimate). The new culvert will also lower the current water level, which the proponents claim will provide new riparian habitat for reptiles and amphibians. The Hamel Island culverts will reconnect Lake aux Dorés to its former bay, as no water or fish can currently pass through. As the water level in the bay is much higher than in Lake aux Dorés, replacing the culvert will also lower the bay’s water level which is hoped to reduce Spring flooding, although the lower water levels will also make ~500 m^2^ of spawning habitat in the bay inaccessible. Locals claim fishing in the bay has worsened, and the proponents claim the new culverts will positively affect fish populations through increased gene flow and habitat diversification. However, there is a risk of contamination upon reconnection, as a site (i.e., Merril Island) in Lake aux Dorés is polluted with mine tailings. No physicochemical measurements were taken as the proponent claimed a low risk of contamination since water flows towards the polluted site and because the mine tailings are assumed to not move upstream.

No habitat compensation was explicitly required for the road repair project, as culvert replacement and maintenance was included. DFO considers the effects of culvert installation to be small, even with many culverts, as some required the free movement of fish. In general, DFO predicted the nearby fish habitats to be the same afterwards if the mitigation and restoration measures were properly done during the work. Despite several instances of noncompliance, including a hydrocarbon spill, fish deaths, and sediment input, no compensation was required for these unexpected HADDs. The contractors were informed of these violations until the situation was remedied, and despite being fined, DFO did not require further fish habitat compensation.

*Fish Passage*

For the hydro project, a migratory pass and weir were developed on the Eastmain River. Fish access to the Rupert River tributaries, free movement of fish through the Rupert River upstream migration channels and the Eastmain River fish pass, and free movement of fish during construction of the Sarcelle powerhouse were also monitored. One of the upstream migration channels was not passable, while only the largest fish could cross the other. For the Eastmain River fishway, walleye, northern pike, longnose sucker and white sucker passed through easily, while lake sturgeon and lake whitefish had difficulty passing and will continue to be monitored.

*Free Movement of Fish*

The only evaluated impact of culverts on fish is on the free movement of fish. For the culvert compensation project with BlackRock Mine, efforts were made to ensure water flow through the culverts even in low flow conditions. To do this, they used large stones to create flow pathways within the culverts, which they embedded into the substrate using a shovel. For the hydro project, two permanent upstream migration channels in the Rupert River enable fish passage for numerous fish species located downstream of the hydropower infrastructure.

**References**

Asselin H (2015) Indigenous forest knowledge. In: K. Peh, R. Corlett & Y. Bergeron (eds.), Routledge Handbook of Forest Ecology. New York: Earthscan, Routledge; p. 586-596.
